# Hydrogen Peroxide-Induced Re-Expression of Repressor Element 1-Silencing Transcription Factor Contributes to Cardiac Vagal Dysfunction in Type 2 Diabetes Mellitus

**DOI:** 10.3390/antiox14050588

**Published:** 2025-05-14

**Authors:** Dongze Zhang, Huiyin Tu, Wenfeng Hu, Yu Li, Michael C. Wadman, Yu-Long Li

**Affiliations:** 1 Department of Emergency Medicine, University of Nebraska Medical Center, Omaha, NE 68198, USA; dongze.zhang@uth.tmc.edu (D.Z.); huiyintu@unmc.edu (H.T.);; 2 Department of Cellular & Integrative Physiology, University of Nebraska Medical Center, Omaha, NE 68198, USA

**Keywords:** antioxidant, cardiac vagal neurons, hydrogen peroxide, N-type Ca^2+^ channels, REST, type 2 diabetes mellitus

## Abstract

Diabetes mellitus, especially type 2 diabetes mellitus (T2DM), is a major health problem worldwide and has become a leading cause of mortality. As a common complication of patients with T2DM, cardiac autonomic dysfunction (including sympathetic overactivation and reduced vagal tone) is associated with a higher risk of arrhythmia-related sudden cardiac death. Our previous study found that T2DM-elevated hydrogen peroxide (H_2_O_2_) levels in atrioventricular ganglion (AVG) neurons contribute to the decrease in cardiac vagal function and ventricular arrhythmogenesis through inhibition of N-type Ca^2+^ channels (Ca_v_2.2). In the present study, treatment with exogenous H_2_O_2_ in differentiated NG108-15 cells increased REST expression and decreased Ca_v_2.2-α expression. Adenoviral catalase gene transfection into the AVG neurons significantly reduced the REST levels elevated by a high-fat diet plus streptozotocin-induced T2DM. Lentiviral REST shRNA transfection markedly increased Ca_v_2.2-α expression in the AVG neurons from T2DM rats. REST shRNA also activated N-type Ca^2+^ channels and increased cell excitability of AVG neurons in T2DM rats. Additionally, REST shRNA markedly improved cardiac vagal activation in T2DM rats. The present study suggests that the H_2_O_2_-REST-Ca_v_2.2 channel signaling axis could be a potential therapeutic target to normalize cardiac vagal dysfunction and its related cardiac complications in T2DM.

## 1. Introduction

Diabetes mellitus, especially type 2 diabetes mellitus (T2DM), is a major cause of mortality [1,2,3,4]. Cardiac autonomic dysfunction (including sympathetic overactivation and reduced vagal tone) affects approximately 20% of people with diabetes [5], which is linked to sudden cardiac death related to arrhythmia and high mortality in patients with T2DM [6,7,8,9,10,11,12,13]. About 1% of patients with T2DM have ventricular arrhythmias [14], and long-term follow-up of the Framingham cohort and their offspring reported that about 20% of patients with arrhythmia-related sudden cardiac death had diabetes [15,16,17]. Indeed, increasing cardiac vagal tone has been found to limit cardiac arrhythmia and to reduce mortality [18,19,20,21,22,23]. It has been reported that cardiac vagal function is severely damaged in T2DM patients [24]. Our previous study has demonstrated a decrease in N-type Ca^2+^ channel expression and cell excitability in atrioventricular ganglion (AVG, also named cardiac vagal postganglionic) neurons from T2DM rats [25]. Very recently, we further demonstrated that endogenous hydrogen peroxide (H_2_O_2_) elevation was a major factor affecting N-type Ca^2+^ channel expression/activation and cell excitability in AVG neurons, further reducing cardiac vagal activation and ventricular arrhythmogenesis in T2DM [26]. Until now, it is unclear how the endogenous H_2_O_2_ reduces N-type Ca^2+^ channel expression/activation. Therefore, this study aims to gain further insight into the mechanisms underlying H_2_O_2_-reduced AVG neuronal excitation by inhibiting the N-type Ca^2+^ channel expression/activation in T2DM.

As a zinc finger transcription factor, protein expression of the repressor element 1- silencing transcription factor (REST)/neuron-restrictive silencer factor (NRSF) is found in multipotent neural stem cells and non-neuronal cells [27,28]. REST interacts with the neuron-restrictive silencer element (NRSE/RE-1, a regulatory region of the target genes) to repress gene transcription [29,30,31]. Since many neuron-specific genes contain NRSE/RE-1 sequences and REST is not expressed in mature neuronal cells, the originally proposed role of REST is to transcriptionally silence neuronal gene expression in non-neuronal cells [32,33,34]. Although the role of REST in embryonic neural cells and neural cells during differentiation has been well studied [35,36,37], its roles in adult neurons have been investigated recently [37,38]. Re-expression or overexpression of REST in some mature neurons has been linked to many diverse types of neurological diseases [38,39,40]. Alongside its function as a master regulator of neuronal genes, REST restricts neuronal excitation-related genes to reduce the cell excitability of the neurons [41]. Overexpression of REST resulted in a progressive reduction in the firing frequency of the action potential (AP) by decreasing the density of voltage-gated Na^+^ channels in excitatory neurons [42]. In addition to its modulation in Na^+^ channels [42,43], a functional role of REST in the silencing of the Ca^2+^ channel gene transcription has been reported in the nervous system [44,45]. It has been identified that the *Cacna1b* gene encodes the α-subunits of N-type Ca^2+^ channels, which have the binding site of NRSE/RE-1 [46], and REST blocks the transcription of the *Cacna1b* gene [47]. However, it remains unclear if REST can inhibit neuronal excitability via modulating N-type Ca^2+^ channel expression, especially in the AVG neurons of the T2DM state. Given that reactive oxygen species (ROS) donors dose-dependently trigger REST overexpression [48], the REST signaling pathway possibly contributes to H_2_O_2_-reduced N-type Ca^2+^ expression/activation and cell excitability in AVG neurons of the T2DM state. Based on the information from the aforementioned studies, we hypothesized that H_2_O_2_ induces the re-expression of REST, which reduces N-type Ca^2+^ channel expression/activation and neural excitability of AVG neurons and further blunts the cardiac vagal function in T2DM rats.

## 2. Materials and Methods

This study was approved by the Institutional Animal Care and Use Committee (IACUC) at the University of Nebraska Medical Center (IACUC number: 18-023-04-FC).

### 2.1. T2DM Animal Model

In an environment with controlled temperature and humidity and a 12 h:12 h dark/light cycle, male Sprague-Dawley rats (6–7 weeks of age, 180–200 g, SASCO) were treated with water and rat chow. In this study, 72 rats were randomly assigned to sham (12 rats) and T2DM (60 rats) groups. T2DM was induced as previously described [25,49]. Briefly, after the rats were fed a high-fat diet (42% fat, 42.7% carbohydrates, and 15.2% protein, Harlan Teklad adjusted fat diet; Harlan Teklad, Madison, WI, USA) for four weeks, the rats were intraperitoneally injected with STZ (30 mg/kg) and continued on the high-fat diet. The sham rats were fed a normal chow diet (13% fat, 53% carbohydrates, and 34% protein, Harlan Teklad sterilizable rodent diet; Harlan Teklad, Madison, WI). Fasting blood glucose and body weight in all rats were measured (Table 1). T2DM rats were then divided into 5 subgroups for different interventions, including T2DM, T2DM+Ad.Empty, T2DM+Ad.Catalase, T2DM + Scrambled shRNA, and T2DM+REST shRNA (12 rats/group). At 12–14 weeks of feeding with either a normal chow diet or a high-fat diet, all experiments were performed because our previous studies revealed the impairment of AVG neural function and cardiac vagal dysfunction during this period [25,26].

### 2.2. Microinjection of Adenoviral Catalase Gene or Lentiviral REST shRNA into the Atrioventricular Ganglion (AVG)

Under anesthesia (2% isoflurane, Butler Schein Animal Health, Dublin, OH, USA) and mechanical ventilation, a left posterolateral thoracotomy was performed through the 3rd left intercostal space, and the left lung was moved aside to expose the AVG. The heartbeat was very weak at the junction of the inferior pulmonary veins and left atrium when the AVG was exposed under the left posterolateral thoracotomy, which ensured the success of microinjection into the AVG. Under the microscope, 2 µL of saline, adenoviral catalase gene (Ad.CAT, 1 × 10^10^ pfu/mL, University of Iowa, Iowa City, IA, USA), adenoviral vector control (Ad.Empty, 1 × 10^10^ pfu/mL, University of Iowa, Iowa City, IA, USA), rat lentiviral REST shRNA (Lenti.REST shRNA, 29mer target-specific shRNA designed against multiple slice variants based on NM_031788 and NM_031788.1, 1 × 10^7^ TU/mL, CAT#: TL711581V, OriGene Technologies, Inc., Rockville, MD, USA), or lentiviral scrambled shRNA (Lenti.scrambled shRNA, lenti particles carrying a 29mer scrambled shRNA sequence cassette, 1 × 10^7^ TU/mL, CAT#: TR30021V, OriGene Technologies, Inc., Rockville, MD, USA) was microinjected into the AVG by a glass micropipette connected to a WPI Nanoliter 2000 microinjector (World Precision Instruments, Sarasota, FL, USA). After one week of gene or shRNA microinjection, the experiments were performed. Regarding the efficiency of adenovirus and lentivirus transfection, our previous studies showed that adenoviral GFP and lentiviral GFP were expressed in all AVG neurons when they were microinjected in the AVG [26,50].

### 2.3. Measurements of Vagal Control of Ventricular Function

Vagal control of ventricular function was determined, as described previously [50,51]. Briefly, under the anesthesia and artificial ventilation, a Millar pressure transducer (SPR 524; size, 3.5-Fr; Millar Instruments, Houston, TX, USA) was put into the left ventricle through the right carotid artery to test left ventricular systolic pressure (LVSP) and the maximum rate of left ventricular pressure rise (LV dP/dt_max_) under the left cervical vagal nerve stimulation applied by a Grass S9 stimulator (Grass instruments, Quincy, MA, USA) (0.1 ms pulse duration and intensity of 7.5 V at 1–100 Hz). Left vagal efferent nerve-stimulated changes of LVSP and LV dP/dt_max_ were recorded by PowerLab 8/30 data acquisition system with LabChart software version 7 (ADInstruments, Colorado Springs, CO, USA), which serves as the index of vagal control of ventricular function.

### 2.4. Isolation of AVG Neurons, and Whole-Cell Patch-Clamp Recording of Ca^2+^ Currents and APs

At the terminal experiment, the AVG was exposed, and neurons were isolated by a 2-step enzymatic digestion protocol, as described previously [25,50,51,52]. The first step was to incubate the minced AVG with a modified Tyrode solution containing 0.1% collagenase and 0.1% trypsin for 30 min at 37 °C. The second step was to transfer the tissue to a modified Tyrode solution containing 0.2% collagenase and 0.5% bovine serum albumin for 30 min of incubation at 37 °C. Finally, the isolated neurons were cultured at 37 °C in a humidified atmosphere of 95% air–5% CO_2_ for patch-clamp experiments, as described previously [25,50,51,52].

Voltage-gated Ca^2+^ currents and APs were recorded in isolated AVG neurons by the whole-cell patch-clamp technique using an Axopatch 200B patch-clamp amplifier (AutoMate Scientific, Inc., Berkeley, CA, USA) [25,50,51,52,53]. In the voltage-clamp measurement, peak Ca^2+^ currents were measured for each test potential. ω-Conotoxin GVIA (Alomone Labs, Ltd., Jerusalem, Israel), a specific N-type Ca^2+^ channel blocker, was used to block the N-type Ca^2+^ channel, and N-type Ca^2+^ currents were obtained by subtracting Ca^2+^ currents under treatment of ω-conotoxin GVIA from total Ca^2+^ currents. Current density was calculated by dividing peak current by cell membrane capacitance. In current-clamp experiments, AP was recorded during a 1 s current clamp under a current injection of 100 pA. Data acquisition and analysis were performed by the P-clamp 10.2 program (AutoMate Scientific, Inc., Berkeley, CA, USA). All experiments were performed at room temperature (22–24 °C).

### 2.5. Reverse-Phase Protein Array (RPPA)

Considering small AVG samples (1–2 mg wet weight), we used a modified reverse-phase protein microarray to measure protein expression in the AVG, as previously described [50,54]. Briefly, a bicinchoninic acid protein assay kit (Pierce, Rockford, IL, USA) was used to measure the total protein concentration in the supernatant of the AVG. Fifty nanoliters of samples were dropped onto nitrocellulose-coated glass slides by an 8-pin arrayer. All samples were then sequentially incubated with primary antibodies (rabbit anti-catalase antibody, #14097s, Cell Signaling; rabbit anti-REST antibody, BS-2590R, Bioss Inc.; rabbit anti-CACNA1B antibody, #ACC-002, Alomone Labs; and mouse anti-β-actin antibody, Sc-4778, Santa Cruz Biotechnology, Inc., Dallas, TX, USA) and LI-COR fluorescence-conjugated secondary antibodies (IRDye 800CW goat anti-rabbit IgG and IRDye 680LT goat anti-mouse IgG). The protein signals were scanned with a LI-COR Odyssey IR imaging system (LI-COR, Lincoln, NE, USA). ImageJ analysis software version 1.53v (NIH, Bethesda, MD, USA) was used to quantify protein expression.

### 2.6. NG108-15 Cell Culture and Treatment of H_2_O_2_ in Differentiated Cells

NG108-15, a hybrid of mouse neuroblastoma N18TG2 and rat glioma C6-BU-1 cell lines, was obtained from the American Type Culture Collection (ATCC, Manassas, VA, USA). Cultured NG108-15 cells were differentiated by a serum-free medium consisting of DMEM, N2 supplements, 1 mM dBcAMP, and antibiotics. Cells with 5 days of differentiation were used for experiments because our previous study found that the Ca_v_2.2-α protein is significantly expressed in NG108-15 cells after 5-day differentiation [55].

**Figure 1:**
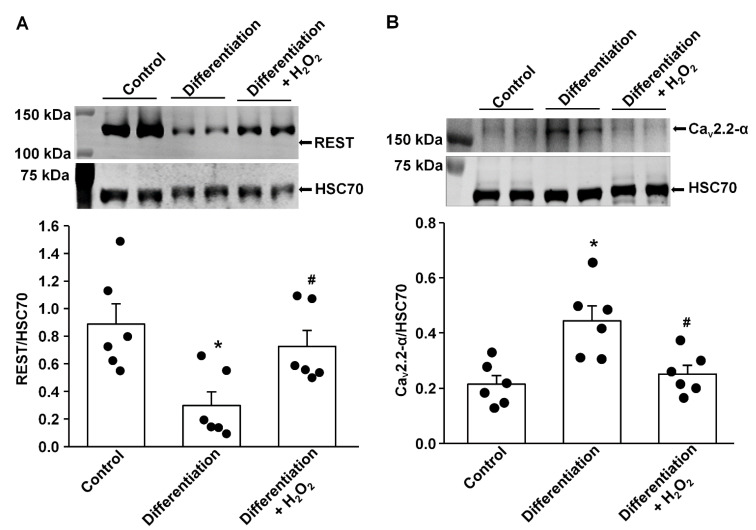
Effect of H_2_O_2_ on protein expression of REST and N-type Ca^2+^ channels (Ca_v_2.2-α) in differentiated NG108-15 cells. (**A**) Raw and quantitative data representing the REST protein levels in undifferentiated NG108-15 cells (Control), 5-day differentiated NG108-15 cells, and 5-day differentiated NG108-15 cells treated with H_2_O_2_ for 24 h, measured by Western blot analysis. (**B**) Raw and quantitative data representing Ca_v_2.2-α protein levels in undifferentiated NG108-15 cells (Control), 5-day differentiated NG108-15 cells, and 5-day differentiated NG108-15 cells treated with H_2_O_2_ for 24 h. Black dots represent each individual data point. *N* = 6 measurements/group. Data are means ± SEM. One-way ANOVA with post-hoc Bonferroni test was used to assess statistical significance. * *p* < 0.05 vs. Control; ^#^ *p* < 0.05 vs. 5-day differentiation.

Although we measured cytosolic H_2_O_2_ production in AVG neurons using H_2_O_2_-specific indicators [26], we could not know the absolute level of intracellular H_2_O_2_ in sham and T2DM rats. One previous study reported that 1–10 nM of intracellular H_2_O_2_ and 0.1–100 μM of extracellular H_2_O_2_ could trigger redox signaling as the oxidative stress [56]. Therefore, we selected 100 nM H_2_O_2_ as the oxidative stress in the present study to trigger the expression of REST in differentiated NG108-15 cells (Figure 1).

### 2.7. Western Blot Analysis

Western blot was performed as previously described [55]. Briefly, a bicinchoninic acid protein assay kit (Thermo Fisher Scientific, Rockford, IL, USA) was used to test total protein concentration in the supernatant of NG108-15 cell lysates. All protein samples (20 μg/well) were loaded on a 9% sodium dodecyl sulfate (SDS)-polyacrylamide gel and then transferred onto a PVDF membrane (EMD Millipore, Billerica, MA, USA). The transferred membrane was successively probed with rabbit primary antibody against REST (BS-2590R, Bioss Inc., Woburn, MA, USA) or rabbit primary antibody against Ca_v_2.2-α (Alomone Labs, Jerusalem, Israel) overnight at 4 °C, and peroxidase-conjugated appropriate secondary antibody (Thermo Fisher Scientific, Rockford, IL, USA). The same membrane was reprobed with mouse anti-HSC 70 antibody (sc-7298, Santa Cruz Biotechnology, Dallas, TX, USA). A UVP bioimaging system (UVP, Upland, CA, USA) was used to analyze all bands. The target protein was normalized by HSC 70 (a housekeeping protein).

### 2.8. Statistical Analysis

All data are presented as means ± SEM. SigmaPlot version 12 was used for data analysis. The Kolmogorov-Smirnov test and equal variance with Levene’s test were used to confirm the normal distribution of the data. One-way ANOVA with post-hoc Bonferroni test was used to assess statistical significance for RPPA, patch-clamp, and Western blot data. Two-way repeated measures ANOVA with post-hoc Bonferroni was used to test statistical significance for vagal control of ventricular function. Statistical significance was accepted when *p* < 0.05.

## 3. Results

### 3.1. Effect of H_2_O_2_ on REST Expression In Vitro and In Vivo

**Figure 2:**
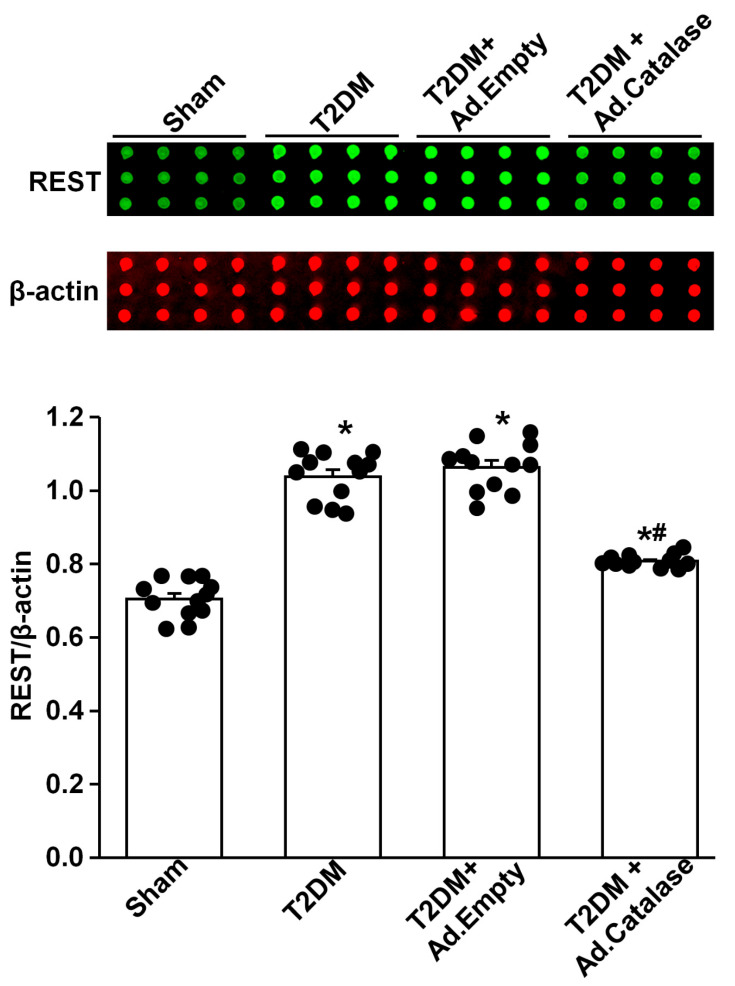
Effect of adenoviral catalase (Ad.CAT) gene transfection on REST protein expression in AVG neurons from T2DM. Raw images (top panel) and quantitative data (bottom panel) representing the REST protein levels in AVG neurons from all groups of rats, analyzed by reverse-phase protein microarray. Black dots represent each individual data point. *N* = 12 measurements from 6 rats/group. Data are means ± SEM. One-way ANOVA with post-hoc Bonferroni test was used to assess statistical significance. * *p* < 0.05 vs. sham; ^#^ *p* < 0.05 vs. T2DM.

Our previous study demonstrated that endogenous H_2_O_2_ elevation inhibited the expression/activation of N-type Ca^2+^ channels and reduced neuronal excitability in AVG neurons from T2DM rats [26]. To advance our understanding of the molecular mechanisms of T2DM-decreased N-type Ca^2+^ channel expression/activation, we evaluated whether REST expression can be directly modulated by oxidative stress. After NG105-15 cells (a cholinergic neuronal cell line) were differentiated for 5 days, the protein expression of REST was decreased, whereas the expression of Ca_v_2.2-α protein was enhanced, compared with non-differentiated (control) cells (Figure 1). When differentiated NG108-15 cells were treated with H_2_O_2_ (100 nM) for 24 h, exogenous H_2_O_2_ markedly increased REST expression and reduced Ca_v_2.2-α expression in differentiated NG108-15 cells (Figure 1). Additionally, T2DM induced the elevation of REST protein in AVG neurons, compared to sham rats (Figure 2). More importantly, when the Ad.CAT gene (an endogenous antioxidant) was *in vivo* transfected into AVG neurons, it partially decreased REST protein expression in the AVG from T2DM rats. However, Ad.empty transfection did not change the expression of the REST protein in T2DM AVG neurons (Figure 2). These data suggest that H_2_O_2_ is a key factor contributing to the overexpression of REST, and the latter could be associated with the reduction of Ca_v_2.2-α protein expression in the AVG neurons of T2DM rats. Our next experiments directly evidence whether overexpression of REST subsequently represses Ca_v_2.2-α protein expression in the AVG neurons from T2DM rats.

### 3.2. Effect of REST on Protein Expression of N-Type Ca^2+^ Channels

**Figure 3:**
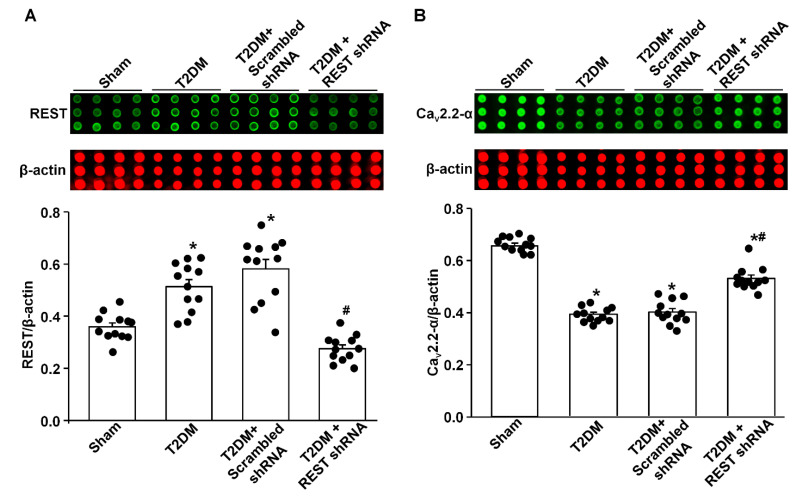
Effect of REST shRNA transfection on N-type Ca^2+^ channel (Ca_v_2.2-α) protein expression in AVG neurons from T2DM rats. (**A**) Raw and quantitative data representing the REST protein levels in AVG neurons from all groups of rats, analyzed by reverse-phase protein microarray. (**B**) Raw and quantitative data representing Cav2.2-α protein levels in the AVG neurons from all groups of rats. Black dots represent each individual data point. *N* = 12 measurements from 6 rats/group. Data are means ± SEM. One-way ANOVA with post-hoc Bonferroni test was used to assess statistical significance. * *p* < 0.05 vs. sham; ^#^ *p* < 0.05 vs. T2DM.

Like the above experiments, there was a low-level expression of REST in the AVG from sham rats, while REST protein expression was significantly increased in the AVG from T2DM rats (Figure 3A). *In vivo* transfection of Lenti.REST shRNA into the AVG markedly reduced REST protein expression in the AVG from T2DM rats (Figure 3A). Additionally, this transfection of REST shRNA also partially increased Ca_v_2.2-α protein expression in AVG neurons from T2DM rats (Figure 3B). However, the transfection of scrambled shRNA did not change the expression of REST and Ca_v_2.2-α proteins in the AVG from T2DM rats (Figure 3).

### 3.3. Effect of REST on N-Type Ca^2+^ Currents and Cell Excitability of AVG Neurons in T2DM

**Figure 4:**
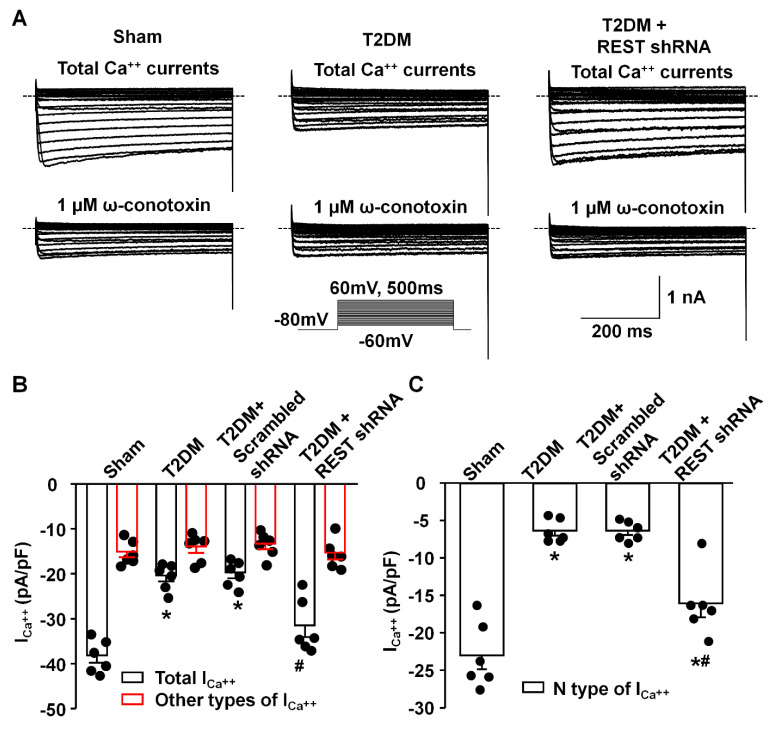
Effect of REST shRNA transfection on N-type Ca^2+^ currents in AVG neurons from T2DM rats. (**A**) Raw data for Ca^2+^ currents from sham, T2DM, and T2DM + lenti.REST shRNA rats. (**B**) Quantitative data for total Ca^2+^ currents and other types of Ca^2+^ currents (cells treated with ω-conotoxin GVIA, a specific N-type Ca^2+^ channel blocker) recorded under the test pulse at 0 mV in AVG neurons from all groups of rats. (**C**) Quantitative data for N-type Ca^2+^ currents analyzed from the original recording in AVG neurons from all groups of rats. Subtracting Ca^2+^ currents under treatment of ω-conotoxin GVIA from total Ca^2+^ currents in the original recording was used to obtain N-type Ca^2+^ currents. Black dots represent each individual data point. *N* = 6 neurons from 4 rats/group; data are means ± SEM. One-way ANOVA with post-hoc Bonferroni test was used to assess statistical significance. * *p* < 0.05 vs. sham; ^#^ *p* < 0.05 vs. T2DM.

**Figure 5:**
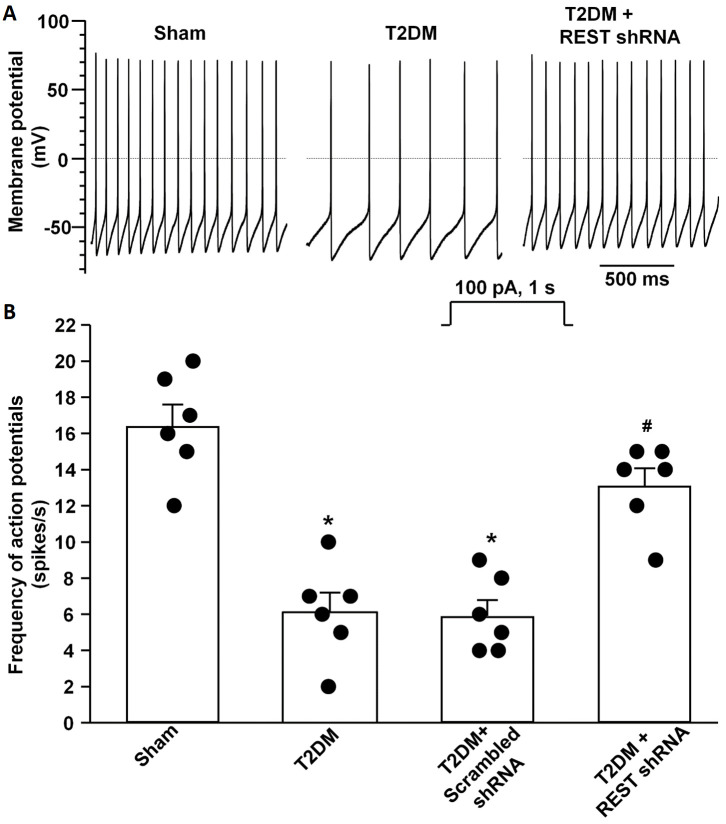
Effect of REST shRNA transfection on neuronal excitability in AVG neurons from T2DM rats. Raw (**A**) and quantitative data (**B**) for action potentials during a 1 s current clamp with a current injection of 100 pA in AVG neurons from all groups of rats. Black dots represent each individual data point. *N* = 6 neurons from 4 rats/group; data are means ± SEM. One-way ANOVA with post-hoc Bonferroni test was used to assess statistical significance. * *p* < 0.05 vs. sham; ^#^ *p* < 0.05 vs. T2DM.

By whole-cell patch-clamp recording, we analyzed the alterations of voltage-gated Ca^2+^ currents and neuronal excitability. Compared to sham rats, total Ca^2+^ currents and N-type Ca^2+^ currents were reduced, whereas other types of Ca^2+^ currents did not change in AVG neurons from T2DM rats (Figure 4), which is consistent with our previous studies [25,26]. *In vivo* transfection of REST shRNA corrected T2DM-reduced total Ca^2+^ currents and N-type Ca^2+^ currents but did not change other types of Ca^2+^ currents in AVG neurons (Figure 4). Additionally, the transfection of REST shRNA also normalized T2DM-reduced cell excitability (frequency of APs) of AVG neurons (Figure 5). No alterations of Ca^2+^ currents and frequency of APs in T2DM AVG neurons after *in vivo* transfection of scrambled shRNA were observed (Figure 4 and Figure 5).

### 3.4. REST shRNA Improved Vagal Control of the Ventricular Function in T2DM

**Figure 6:**
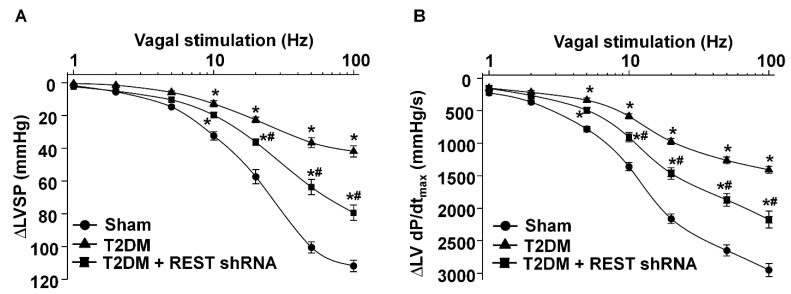
Effect of REST shRNA on vagal control of the ventricular function in T2DM. Left vagal efferent nerve stimulation (VNS)-induced changes of the left ventricular systolic pressure (LVSP, panel **A**) and the maximum rate of increase of left ventricular pressure (LV dP/dt_max_, panel **B**) represented the vagal control of the ventricular function. *N* = 6 rats/group; data are means ± SEM. Two-way repeated measures ANOVA with post-hoc Bonferroni was used to test statistical significance. * *p* < 0.05 vs. sham; ^#^ *p* < 0.05 vs. T2DM.

As an index of ventricular vagal activation, the vagal efferent nerve-triggered negative ventricular inotropy was evaluated. T2DM significantly attenuated ventricular vagal function, compared to the sham group (Figure 6). REST shRNA *in vivo* transfected into AVG neurons significantly restored T2DM-blunted changes of the vagal efferent nerve-stimulated LVSP and LV dP/dt_max_ (Figure 6). Reduced expression/activation of N-type Ca^2+^ channels in AVG neurons attenuated vagal control of ventricular function [50]. Combining our previous studies with the present study, we further confirm that REST elevation-reduced N-type Ca^2+^ channel expression/activation could contribute to the ventricular vagal dysfunction in T2DM.

## 4. Discussion

Our current study reported a significant contribution of REST to T2DM-induced AVG neuronal dysfunction and withdrawal of cardiac vagal activity. We demonstrated for the first time that the protein expression of REST was high, whereas N-type Ca^2+^ channel expression was low in the AVG from T2DM rats (Figure 3), which was also confirmed in differentiated NG108-15 cells (a cholinergic neuronal cell line, Figure 1). Inhibition of REST expression by *in vivo* transfection of REST shRNA in the AVG markedly restored T2DM-reduced N-type Ca^2+^ channel expression/activation with neuronal excitability in AVG neurons (Figure 3, Figure 4 and Figure 5). Our data also demonstrated that treatment with exogenous H_2_O_2_ in differentiated NG108-15 cells markedly increased REST expression and decreased Ca_v_2.2-α expression *in vitro* experiments (Figure 1), whereas Ad.CAT gene *in vivo*-transfected into AVG neurons attenuated REST protein expression (Figure 2) and increased Ca_v_2.2-α expression in the AVG from T2DM rats [26]. More importantly, REST shRNA improved ventricular vagal function in T2DM rats, evidenced by an increase in vagal efferent nerve-stimulated ventricular contractile function. These data suggest that H_2_O_2_-induced re-expression of REST decreases N-type Ca^2+^ channel expression/activation in AVG neurons and subsequently attenuates cardiac vagal function in T2DM.

As a transcriptional repressor, REST interacts with NRSE/RE-1 sequences to down-regulate neuronal gene transcription in embryonic and neural stem cells and in neural cells during differentiation [34,36,57,58]. To date, over two thousand target genes could be repressed by REST in neuronal and non-neuronal cells [27,38,59]. Many factors, including the interactive ability, the availability of the specific DNA binding sites, and the competition with other transcription factors, can influence the down-regulatory role of REST in target gene transcription [60,61,62]. Since REST is not expressed in mature neuronal cells under normal conditions, it was originally proposed that REST is a key transcriptional factor to silence neuronal gene transcription in non-neuronal tissues [32,33,34]. However, growing evidence has demonstrated its role in adult neurons [37,38]. Re-expression of REST in mature neurons has been identified in some neurological diseases [38,39,40]. Although our previous study demonstrated that endogenous H_2_O_2_ elevation-reduced N-type Ca^2+^ channel expression/activation plays a critical role in regulating the cell excitability of AVG neurons in T2DM [26], the molecular mechanisms responsible for H_2_O_2_-inhibited N-type Ca^2+^ channel expression/activation in T2DM remain poorly understood. Since the binding site of NRSE/RE-1 has been identified in *Cacna1b* (a gene encoding Ca_v_2.2-α α-subunit) [46], it is possible that H_2_O_2_-reduced Ca_v_2.2 channel expression/activation might be achieved by REST binding to the *Cacna1b* gene in the T2DM state. To validate this possibility, our present study found that H_2_O_2_ triggered the overexpression of REST in *in vitro* and *in vivo* studies (Figure 1 and Figure 2). Additionally, we also found low expression of REST in the sham AVG and an increase in REST expression in the AVG from T2DM rats (Figure 3). To further evaluate the regulatory role of REST in the expression of N-type Ca^2+^ channels, we *in vivo*-transfected REST shRNA into AVG to knock down REST expression in AVG neurons in T2DM rats. Our data demonstrated that inhibition of REST expression by transfection of REST shRNA into AVG resulted in increased Ca_v_2.2-α expression/activation in AVG neurons from T2DM rats (Figure 3 and Figure 4). These data suggest that up-regulated REST repressor function inhibits N-type Ca^2+^ channel expression in AVG neurons, thereby leading to reduced cell excitability of AVG neurons in T2DM (Figure 3, Figure 4 and Figure 5). Our findings in the present study are supported by other previous studies, in which glutamatergic agonist- or ischemic insults-increased REST decreased expression of neuron-specific genes in mature neurons [63,64,65,66,67].

As mentioned above, REST is a key transcriptional factor to silence neuronal gene transcription in non-neuronal tissues [32,33,34]. REST is also expressed in cardiomyocytes to modulate cardiac structure and function in healthy and pathophysiological conditions [68]. Therefore, the regulatory role of REST in the AVG neurons and cardiomyocytes could contribute to cardiac complications (such as cardiac arrhythmias and sudden cardiac death) in T2DM.

In addition to N-type Ca^2+^ channels, the regulatory role of REST in other ion channels has been widely studied in different physiological and pathophysiological conditions. Overexpression of REST induces a significant reduction in the firing frequency of APs by decreasing the density of voltage-gated Na^+^ channels in excitatory neurons [42]. Additionally, REST has a functional role in regulating the expression of the K^+^ channel in the nervous system [39,69,70,71]. Uchida et al. also demonstrated that REST inhibits K_v_4.3 channel expression, which is involved in the development and maintenance of neuropathic pain [72]. Similar down-regulatory mechanisms were also observed in the *Scn10a* gene (a protein-coding gene for sodium voltage-gated channel 1.8 subtype) in the dorsal root ganglion, in which expression of REST was increased after nerve injury [73]. REST causes the primary sensory nerve C-fiber pathological process through specifically binding to the NRSE/RE-1 within the promoter region of the *Scn10a* gene for epigenetic silencing of Na^+^ channel expression [73]. These published data increased the possibility that REST re-expression-reduced cell excitability of AVG neurons might be achieved not only by repressing N-type Ca^2+^ channels but also by down-regulating Na^+^ and K^+^ channels. Therefore, future studies are required to investigate whether T2DM inhibits the expression of Na^+^ and K^+^ channels in AVG neurons through the re-expression of REST.

Although the binding site of NRSE/RE-1 has been identified in genes encoding other types of voltage-dependent calcium channels (VDCCs), such as R-, P/Q-, and T-type Ca^2+^ channels [45], our previous study demonstrated that T2DM only reduced N-type Ca^2+^ channel expression/activation but did not affect other types of VDCCs [25]. More importantly, in our present study, *in vivo* transfection of REST shRNA corrected T2DM-reduced total Ca^2+^ currents and N-type Ca^2+^ currents but did not change other types of Ca^2+^ currents in AVG neurons (Figure 4). Additionally, the absence of the T-type Ca^2+^ currents in the rat AVG neurons was reported by Xu et al. [74]. Therefore, why the re-expression of REST does not down-regulate the expression/activation of other types of VDCCs should be explored in future studies.

Oxidative stress-related ROS overproduction plays a vital role in the pathogenesis of T2DM [75]. To advance our understanding of the mechanisms responsible for the T2DM-elevated REST expression, we assessed whether elevated oxidative stress contributes to the overexpression of REST in AVG neurons from T2DM rats. Our *in vitro* experiments demonstrated that differentiation of NG108-15 cells for 5 days induced a decrease in REST protein expression with an increase in Ca_v_2.2-α protein expression, whereas treatment with exogenous H_2_O_2_ markedly reversed differentiation-decreased REST protein expression and differentiation-enhanced Ca_v_2.2-α protein expression in NG108-15 cells (Figure 1). Our *in vivo* studies showed that the Ad.CAT gene transfected into AVG neurons significantly decreased REST protein expression (Figure 2) and increased Ca_v_2.2-α protein expression in the AVG from T2DM rats [26]. Additionally, treatment with ROS donors (including H_2_O_2_ and superoxide donors) has been reported to regulate the expression of REST in a dose-dependent manner in human endothelial cells [48]. So far, the role of other oxidative stress-related molecules in the expression of REST and potential molecular mechanisms has not been reported. However, it is very difficult to distinguish the signaling pathways among H_2_O_2_, superoxide, and other oxidative stress-related molecules because these molecules are rapidly converted into other oxidative stress-related molecules by their related enzymes. For example, superoxide is rapidly dismutated into H_2_O_2_ by superoxide dismutase [76,77,78]. Therefore, these data suggest that oxidative stress-related ROS overproduction (including H_2_O_2_, superoxide, and/or other oxidative stress-related molecules) is a key factor to induce REST overexpression in AVG neurons, which subsequently results in the down-regulation of N-type Ca^2+^ channel expression/activation and neuronal excitability of AVG neurons in T2DM.

Since mature neurons are sensitive and easily damaged by high levels of REST [42], we transfected REST shRNA into the AVG to knock down REST expression in AVG neurons and observe its protective role on cardiac vagal function in T2DM rats. Our data demonstrated that REST shRNA transfected into AVG neurons markedly restored the neuronal excitability and subsequently improved the cardiac vagal function in T2DM rats (Figure 5 and Figure 6). From these data, we thought that the inhibition of the REST signaling pathway could be developed into clinical therapy to restore T2DM-reduced cardiac vagal function and improve the outcomes of patients with T2DM. To date, many chemical compounds focusing on restoring the homeostasis of REST could serve as potential candidates for REST-related AVG neuronal dysfunction. For example, X5050, a REST inhibitor, promotes REST degradation to increase some neuronal gene transcriptions [79]. Additionally, as a competitive antagonist of REST, REST-VP16 directly activates gene transcription through binding to the DNA binding site, similar to REST [80]. Furthermore, antioxidant-inhibited REST expression (Figure 2) should be a developing therapeutic strategy for improving cardiac vagal function in T2DM.

In the present study, there was no significant difference in blood glucose levels between the T2DM alone group and T2DM with each intervention group (Table 1). Two possibilities could explain this phenomenon: one is that the H_2_O_2_-REST signaling pathway might be downstream of T2DM-elevated blood glucose, and another is that local treatment with catalase or REST shRNA into the AVG could not affect blood glucose levels. Additionally, although a high-fat diet plus STZ injection induced the frank T2DM in rats characterized by hyperglycemia, hyperlipidemia, and insulin resistance [25], we found that body weight had no significant increase, compared to sham animals (Table 1). It is unclear what causes this phenomenon, but one possibility for this phenomenon is that elevated serum leptin [25] could be associated with a decreased appetite and further affect an increase in body weight in T2DM rats.

## 5. Conclusions

Our study demonstrates that REST expression with its repressor function is up-regulated by oxidative stress, resulting in the down-regulation of N-type Ca^2+^ channel expression/activation in AVG neurons from T2DM rats. Consequently, N-type-Ca^2+^ channel dysfunction reduces neuronal excitability of AVG neurons and blunts cardiac vagal activation in T2DM. These data suggest that the H_2_O_2_-REST-Ca_v_2.2 channel signaling axis could be a potential therapeutic target to normalize cardiac vagal dysfunction and reduce the mortality in T2DM. We understand it is a big challenge to deliver catalase genes or REST shRNAs locally with a viral delivery system in clinical management. Therefore, the development of antioxidants and REST inhibitors as therapeutics might improve cardiac vagal function and prognosis for patients with T2DM.

## Figures and Tables

**Table 1:** Metabolic characteristics in all groups of rats.

	Body Weight (g)	Blood Glucose (mg/dL)
Sham	407 ± 7.7	99.7 ± 3.5
T2DM	419 ± 6.7	437.5 ± 17.8 *
T2DM + Ad.Empty	412 ± 6.7	440.5 ± 15.8 *
T2DM + Ad.Catalase	413 ± 6.6	430.2 ± 10.9 *
T2DM + Scrambled shRNA	412 ± 7.8	443.8 ± 17.7 *
T2DM + REST shRNA	411 ± 7.9	440.2 ± 15.2 *

Data are means ± SEM and *n* = 12/group. One-way ANOVA with post-hoc Bonferroni test was used to assess statistical significance. * *p* < 0.05 vs. sham.

## Data Availability

All data are contained within the manuscript. The original contributions presented in this study are included in the article. Further inquiries can be directed to the corresponding author.
